# Interactive effect of potassium and cadmium on growth, root morphology and chlorophyll *a* fluorescence in tomato plant

**DOI:** 10.1038/s41598-021-84990-4

**Published:** 2021-03-08

**Authors:** Rachida Naciri, Meryeme Lahrir, Chahinez Benadis, Mohamed Chtouki, Abdallah Oukarroum

**Affiliations:** University Mohammed VI Polytechnic (UM6P), Lot-660 Hay Moulay Rachid, 43150 Ben Guerir, Morocco

**Keywords:** Plant sciences, Photosynthesis, Plant development, Plant physiology, Plant stress responses

## Abstract

A hydroponic experiment was conducted to evaluate the role of potassium (K) in tomato plant growth exposed to cadmium (Cd) stress. In this work, the effects of three potassium nutrition regimes (155, 232 and 310 ppm of K) combined with Cd at different levels (0, 12 and 25 µM of CdCl_2_) on chlorophyll content index, root and shoot dry weights, root morphology, chlorophyll *a* fluorescence and translocation factor were analyzed. The results showed a negative effect of cadmium, at different concentrations, on all these parameters. However, optimization of K nutrition has shown promising results by limiting the negative effect of Cd. A positive effect of the high concentration of K (310 ppm) was observed on leaf chlorophyll content and chlorophyll a fluorescence compared to 232 and 155 ppm under Cd stress. K supply improved the electron transport at PSI side indicated by the increase in the amplitude of the I–P phase of OJIP transient. Also, K at a concentration of 310 ppm significantly reduced Cd translocation from root to shoot and improved root and shoot growth parameters in the presence of Cd. K supplementation can reduce the negative effect of Cd by improving photosynthesis and promoting chlorophyll synthesis. The optimization of nutrients composition and concentration might be a good strategy to reduce the impact of Cd on plant growth and physiology.

## Introduction

In plants, bioaccumulation of cadmium (Cd) disturbs many morphological, biochemical and physiological mechanisms^1, 2^. Cd induced negative impact on nutrient uptake, plant growth and biomass by decreasing carbon fixation and other visual symptoms^3–5^. It caused also an induction of reactive oxygen species which damage many cellular functions and plant metabolism^6–8^. Several physiological, biochemical or molecular strategies have been involved by plants to alleviate and encounter Cd induced-toxicity. To preserve plant metal homeostasis and cell redox equilibrium, plants activate for instance an antioxidant defence system to protect the cellular plants from Cd induced-toxicity. This antioxidant complex includes enzymatic molecules (such as catalase, ascorbate peroxidase, glutathione reductase, glutathione S-transferase, and glutathione peroxidase) and non-enzymatic antioxidants molecules (such as glutathione, a-tocopherols, phenolic compounds, ascorbate).

It has been reported that Cd interfaces with mineral elements uptake such as Ca^2+^, Mg^2+^, Cu^2+^, Fe^2+^, Mn^2+^, Zn^2+^ and K^+^^9–12^. Indeed, Cd might compete with Fe, Mn, Zn, and Ca transport and consequently decreases the uptake and translocation of these nutrients or can displace for example Zn and Ca and therefore disturbs cellular signaling^2, 13, 14^. Moreover, it has been reported that mineral nutrition was improved when proline was added to Cd stressed plants^15, 16^. Cd uptake, transport and accumulation in the plant is affected by many physical and chemical factors, such as soil type, pH, cation exchange capacity, plant tolerance, etc.^17–19^. To minimize Cd bioaccumulation in plants, it has been reported that Zinc which is an important micronutrient could play this role^20, 21^. This positive role of Zn was due to its competitive ion for Cd absorption from the soil with the help of their chemical similarity^2^. In other study, it has been shown that N supply induced alleviation of Cd accumulation by enhancing photosynthesis and promoting antioxidant enzymes^22–24^. Also, K supply reduced significantly Cd uptake from the medium in *Brassica campestris L.*^25^. The role of potassium in ameliorating plant growth exposed to Cd has been investigated. In previous studies, to encounter Cd accumulation in plants, the forms and rates of potassium fertilizers should be taken into consideration^26–28^. For instance, chen et al.^29^ showed that the application of K fertilizer in the form of K_2_SO_4_ improved the dry weight of wheat and reduced the phytoavailability of Cd and Pb. However, the concentration of K was considered as an important factor to have an optimal plant growth response. Nowadays, few studies have examined the role of K in the alleviation of Cd impact on tomato plant growth and physiology, and the mechanisms involved in these defense processes are little understood.

It is known that K is the most abundant macroelement nutrient in plants and K plays primordial roles in many physiological and biochemical plant machansim such as, protein synthesis, photosynthesis, plant stress alleviation, cation–anion balance^30–32^. In a previous study, the role of K on Cd stress alleviation was evaluated in *G. grandiflora* plant^33^. These authors observed that K supplementation ameliorated plant growth, total soluble protein and proline and activity of antioxidant enzymes. In our study, K and Cd interaction is elucidated. Experiments were conducted to investigate and compare the role of potassium concentrations in the alleviation of Cd impact on root morphology, physiology and translocation factor of tomato plants grown in hydroponic conditions. We hypothesis that the optimization of K nutrition can reduce Cd accumulation, enhance plant growth and maintain photosynthetic electron chain in photosynthesis.

## Material and methods

### Plant material and growth conditions

Tomato seeds (CAMPBELL 33) were germinated in commercial peat substrate in a growth chamber with a day/night cycle of 16/8 h at 25 °C. Irrigation was performed every day by distilled water. After emergence (2-leaf stage), the seedlings were irrigated with a Hoagland nutrient solution at pH 5.6 (N: 242 ppm as KNO_3,_ Ca(NO_3_)_2_∙4H_2_O and NH_4_NO_3_; P: 31 ppm as KH_2_PO_4_; K: 232 ppm as KNO_3_ and KH_2_PO_4_; Ca: 224 ppm as Ca(NO_3_)_2_∙4H_2_O; Mg: 49 ppm as MgSO_4_∙7H_2_O; B: 0.45 ppm as H_3_BO_3_; Cu: 0.02 ppm as CuSO_4_.5H_2_O; Mn: 0.5 ppm as MnCl_2_∙4H_2_O; Mo: 0.0106 ppm as Na_2_MoO_4_. 2H_2_O; Zn: 0.48 ppm as ZnSO_4_.7H_2_O; and Fe: 0.5% of (NH_4_)_5_[Fe(C_6_H_4_O_7_)_2_] used at rate 1 ml/l of nutrient solution) (Hoagland and Arnon, 1950). The nutrient concentration was gradually increased from 10 to 50% ionic strength every 2 days to avoid any risk or stress due to transplantation. After this adaptation phase (first true leaves appeared), uniform twentyeight-day-old plants were selected and transplanted into 4 L plastic pots in a fully concentrated Hoagland and Arnon solution (Hoagland and Arnon, 1950) with three seedlings per pot. The nutrient solution was continuously aerated and changed every 7 days. The hydroponics experiment was organized as a completely randomized design with six replicates. A week later, plants were exposed to three concentrations of Cd (0, 12 and 25 µM of CdCl_2_) with three K levels (155, 232 and 310 ppm as KH_2_PO_4_) for 21 days.

### Chlorophyll content index

Chlorophyll meter (CL-O1, Hansatech instruments) was used to estimate the chlorophyll content index (CCI) in the middle part of the leaf after 14 days of K and Cd exposure.

### Root and shoot dry weights

All treated-plants were dried in an oven at 70 °C for 2 days to determine root and shoot dry weights.

### Roots morphology parameters

Roots morphology and related characteristics (root length, root average diameter, volume and root surface area) were analyzed using the WinRHIZO image analyzing system (Regent Instructions, Quebec, Canada). Roots were carefully spread over a plastic box and scanned using an Epson Perfection LA2400 scanner. Data were digitalized by processing the scanned root images. Root diameter and surface area were analyzed.

### Leaf chlorophyll a fluorescence

Tomato plants kept in dark for 15 min before the chlorophyll *a* fluorescence (CHF) measurements were started. For each treatment, 15 independent measurements were made by Handy PEA^+^ fluorometer (Handy PEA^+^, Hansatech instruments) on the middle leaves of plants. A single strong 1 s light pulse of 3000 μmol s^−1^ m^−2^ (which is an excitation intensity to ensure closure of all PSII reaction centers) provided by an array of six light-emitting diodes (peak 650 nm) was applied on the middle of each leave. ChlF transients were digitized between 10 µs to 1 s by the instrument. Chlorophyll *a* fluorescence measurement is a non-introsive method used for monitoring the state of plant physiology in different treatment conditions. The ChlF OJIP transient showed a polyphasic rise during the first second of illumination and its intensity increases from a minimum fluorescence intensity F_o_ (when all reaction centers were open and all Q_A_ oxidized) to a maximum fluorescence intensity F_M_ (when all reaction centers were closed and all Q_A_ reduced) and with two intermediate steps named J (F_J_) and I (F_I_). In this work, fluorescence parameters related to photosystem I (PSI) were calculated by the JIP-test Eqs.^34–36^.

$${\text{Sm}} = {\text{Area}}/\left( {{\text{F}}_{{\text{M}}} - {\text{F}}_{{\text{o}}} } \right)\;{\text{where}}\;{\text{Area}}\; = \int\limits_{{{\text{F}}_{{\text{o}}} }}^{{{\text{F}}_{{\text{M}}} }} {\left( {{\text{F}}_{{\text{M}}} {-}{\text{F}}_{{\text{t}}} } \right){\text{dt}}}$$

Sm is a function of the number of electrons transported by PS II in the time range from 0 to tFM, the time to reach the maximum fluorescence intensity.

The efficiency with which an electron can move from the reduced intersystem electron acceptors to the PSI end electron acceptors can be expressed as:

$${\text{RE}}_{{\text{o}}} /{\text{ET}}_{{\text{o}}} = {\text{d}}_{{{\text{Ro}}}} = \left( {1 - {\text{V}}_{{\text{I}}} } \right)/\left( {1 - {\text{V}}_{{\text{J}}} } \right)$$

According to JIP-test, relative variable fluorescence V_t_ is expressed as (F_t_ − F_o_)/(F_M_ − F_o_) and this expression can be taken as a measure of the fraction of the primary quinone electron acceptor of PS II in its reduced state [Q_A_^−^ /Q_A (total)_].

RE_o_/RC expressed the electron transport from Q_A_^−^ to the PSI electron acceptors.

### Translocation factors

Translocation factor was used to evaluate the translocation of Cd from root to shoot, this parameter was calculated following equation:

$${\text{Bioaccumulation}}\;{\text{factor}} = {\text{Cd}}\;{\text{concentration}}\;{\text{in}}\;{\text{shoot}}/{\text{Cd}}\;{\text{concentration}}\;{\text{in}}\;{\text{root}}$$

### Statistical analysis

Analysis of variance (ANOVA) was done in SPSS 13.0 (SPSS Inc., USA) to examine the impacts of Cd and K interaction effects on ChlF parameters, dry matter, chlorophyll content, root morphological traits. Differences between treatment means were evaluated by the Student–Newman–Keuls test at a 0.05 probability level.

## Results

### Chlorophyll content index (CCI)

**Figure 1:**
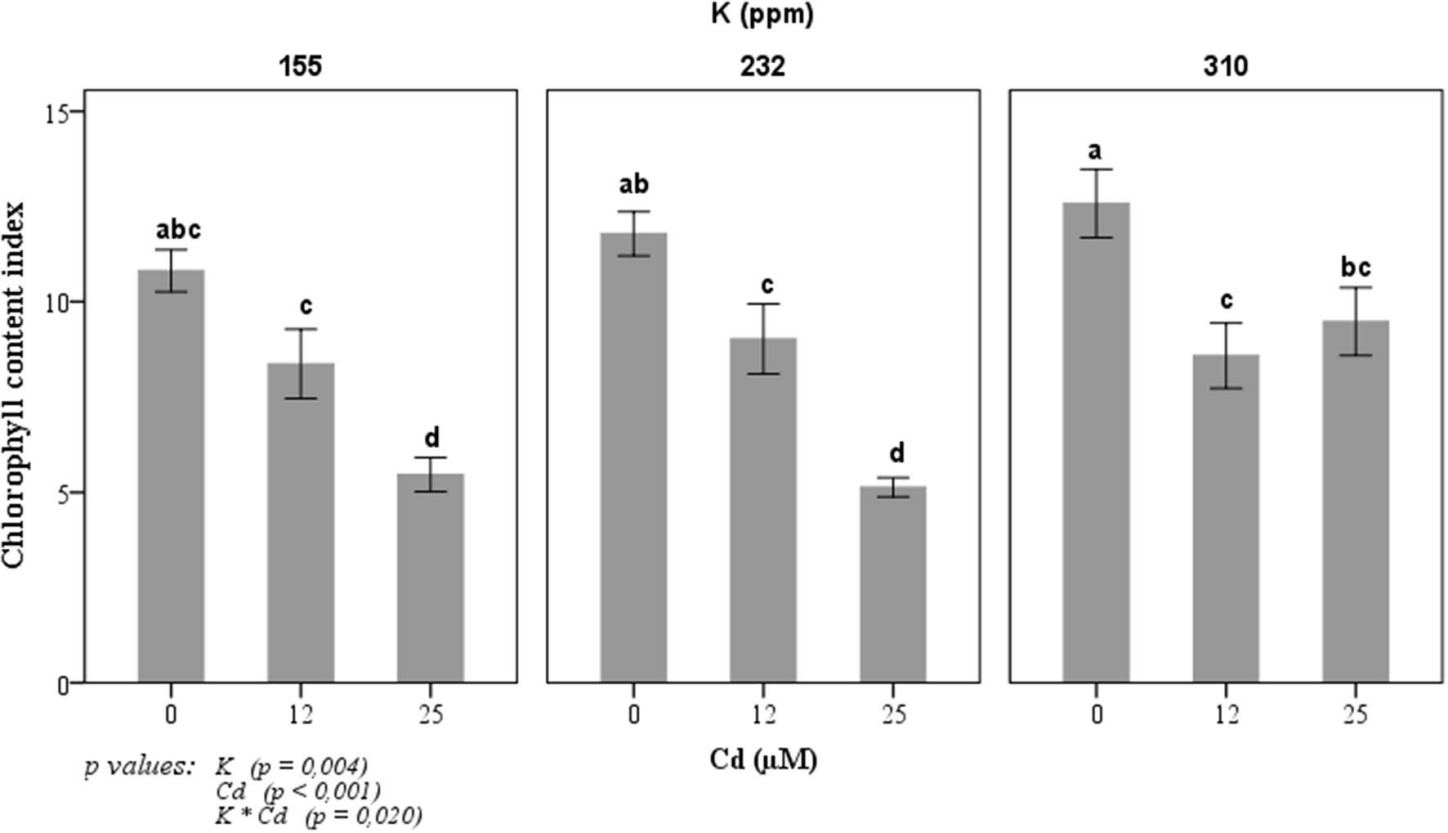
Change in chlorophyll content index of tomato plants in response to three potassium concentrations (155, 232, and 310 ppm) and three cadmium concentrations (0, 12 and 25 µM CdCl_2_). Each value represents the mean ± SD of six independent repetitions, dissimilar letters indicate significant differences at *p* < 0.05 according to Student–Newman–Keuls test.

Chlorophyll content index decreased in tomato leaves when exposed to Cd (Fig. 1). This decrease differed according to the concentration of potassium used in the growth media. This reduction in CCI is significantly observed at a concentration of 25 µM Cd. However, a combination of the concentration of 25 µM Cd and 310 ppm of K has a less negative effect on CCI than those at 155 and 232 ppm of K of the tomato leaves. At 25 µM Cd, CCI decreased by 50, 56 and 25% respectively with 155, 232 and 310 ppm of potassium concentration.

### Plant dry weights

**Figure 2:**
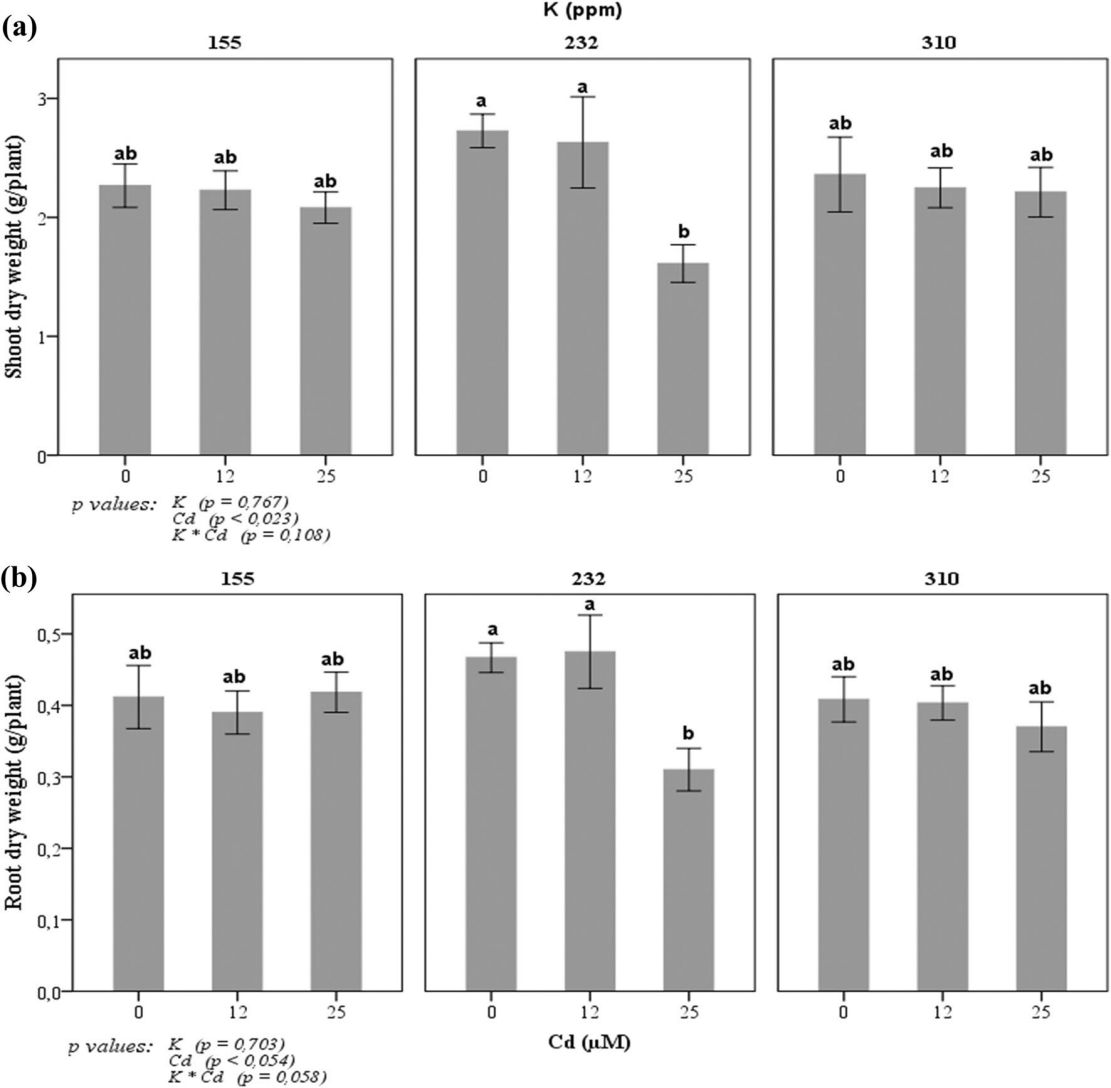
Changes in (**a**) shoot and (**b**) root dry weights of tomato plant in response to three potassium concentrations (155, 232, and 310 ppm) and three cadmium concentrations (0, 12 and 25 µM CdCl_2_). Each value represents the mean ± SD of six independent repetitions, , dissimilar letters indicate significant differences at *p* < 0.05 according to Student–Newman–Keuls test.

The variation of root and shoot dry weight was depended on the applied treatment (Fig. 2). No significant difference in tomato dry weights was observed in 155 and 310 ppm K concentrations. However, a significant variation of these parameters was observed in 232 ppm K treatment. A significant reduction has been noted in plants grown in culture medium with 232 ppm of K combined to 25 µM Cd (− 40%). Results showed that the highest values of the shoot and root dry weights were observed in plants grown in a culture medium with 232 ppm of K and 0 or 12 µM of Cd. Also, improvement of dry weights was observed in plants grown in 310 ppm K and 25 µM Cd compared to plants grown in 232 ppm K and 25 µM Cd (30% in shoot dry weight and 16% in root dry weight).

### Root morphology

**Figure 3:**
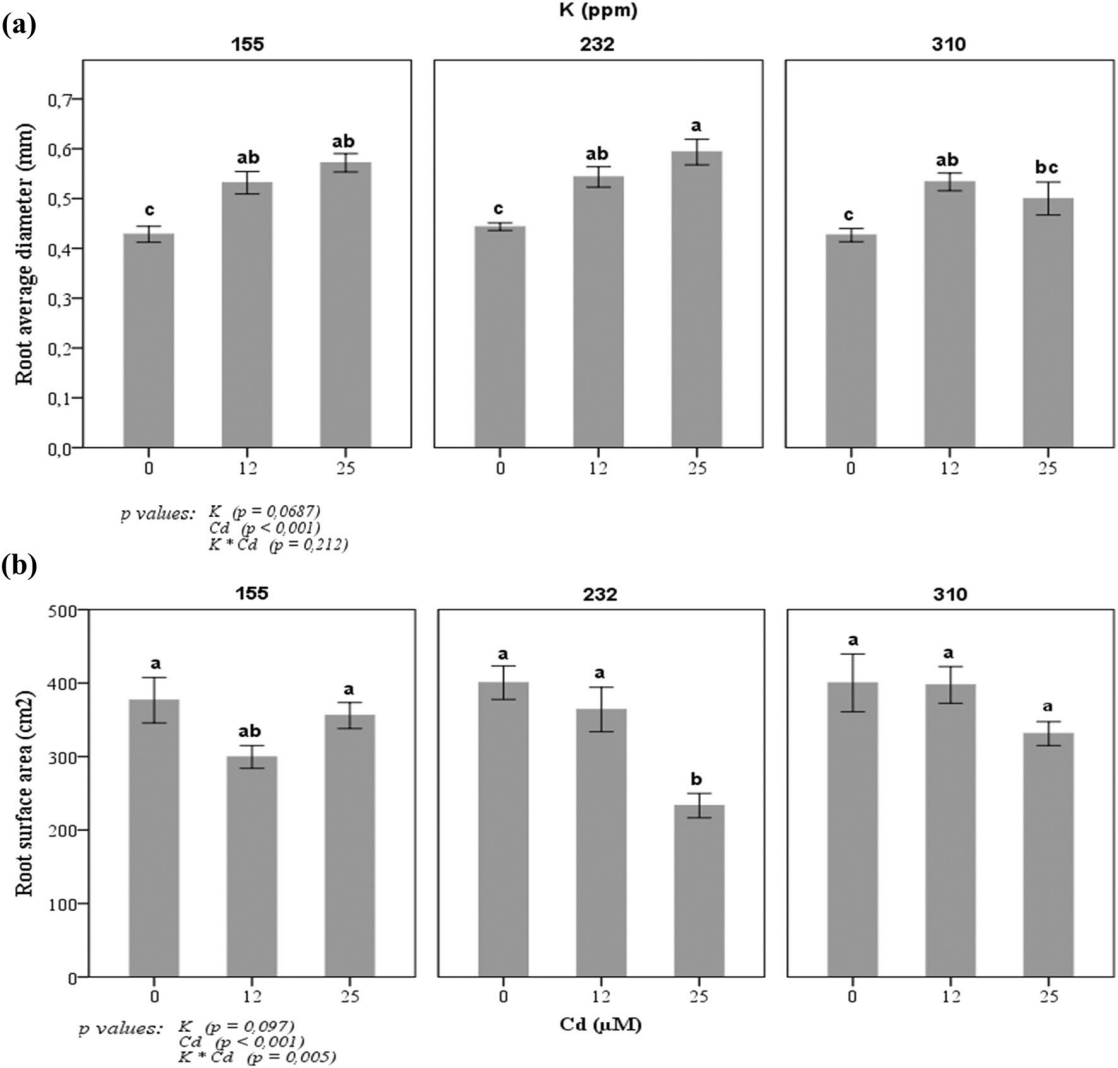
Changes in (**a**) root diameter and (**b**) root surface area (SA) of tomato plant in response to three potassium concentrations (155, 232, and 310 ppm) and three cadmium concentrations (0, 12 and 25 µM CdCl_2_). Each value represents the mean ± SD of six independent repetitions, dissimilar letters indicate significant differences at *p* < 0.05 according to Student–Newman–Keuls test.

The results showed an increase of tomato root diameter in response to Cd stress in all K concentrations compared to plants growing in a medium without Cd (Fig. 3a). In addition, the roots of the plant exposed to Cd appeared to be rigid with visual secondary root development. Also, the root surface area (SA) (Fig. 3b) was affected by K and Cd supply. When the concentration of Cd increased, the SA of the roots decreased, except at treatment with 155 ppm of K. However, in the presence of 310 ppm of K and 25 µM of CdCl_2_, roots SA has been significantly increased by 30% compared to the SA estimated in the roots of plants growing in a growth medium with 232 ppm of K and 25 µM of CdCl_2_.

### Translocation factor of Cd

**Figure 4:**
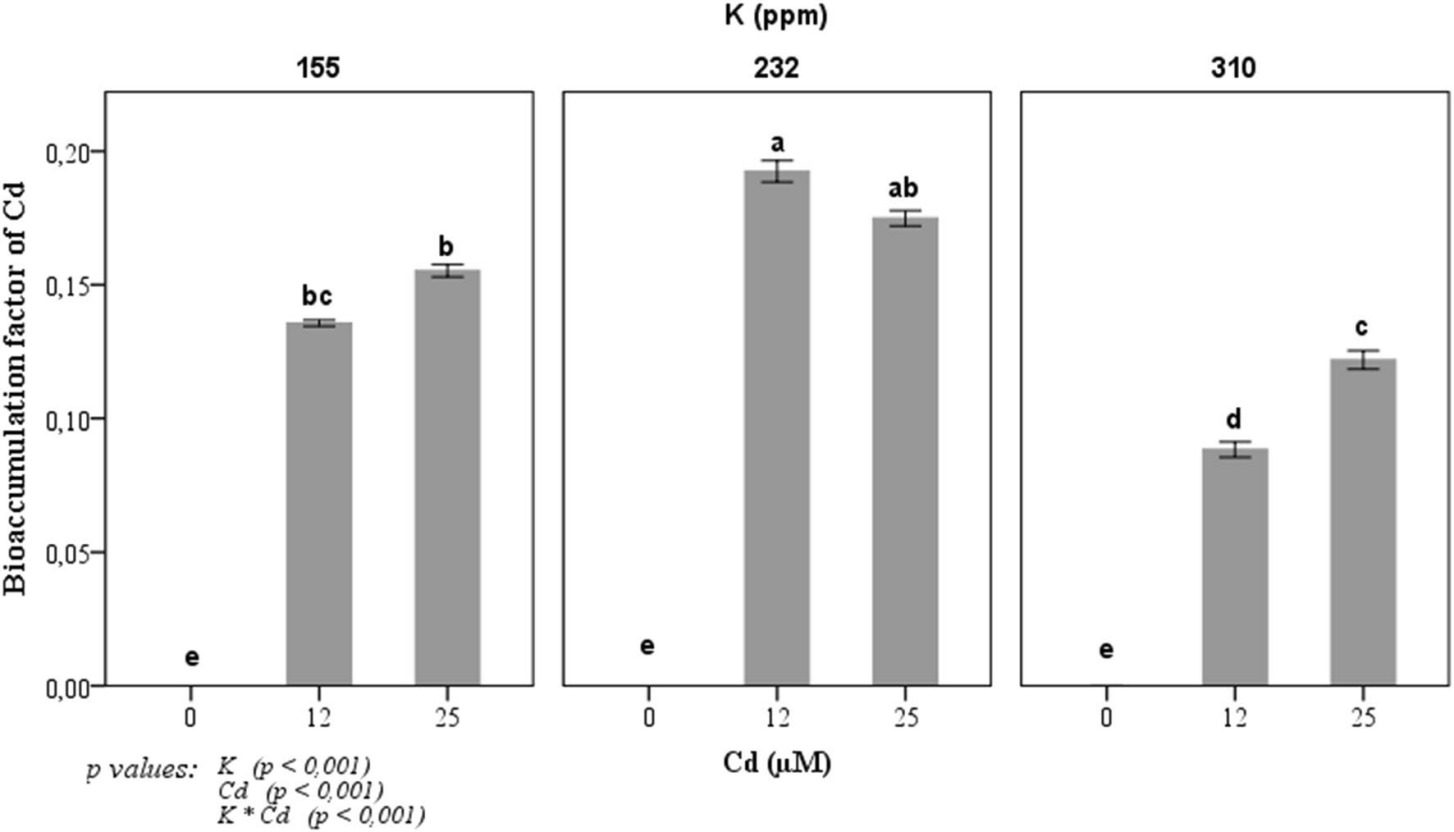
Changes in Cd translocation factor of tomato plant in response to three potassium concentrations (155, 232, and 310 ppm) and three cadmium concentrations (0, 12 and 25 µM CdCl_2_). Each value represents the mean ± SD of six independent repetitions, dissimilar letters indicate significant differences at *p* < 0.05 according to Student–Newman–Keuls test.

In this study, the translocation factor of Cd differs according to the Cd and K concentrations in the culture medium (Fig. 4). The highest Cd concentrations in the growth medium resulted in a significantly high translocation factor. However, the value of this factor was lower in the presence of K. For instance, at a concentration of 25 µM Cd, the translocation factor was 0.15, 0.17 and 0.12 respectively at 155, 232 and 310 ppm of K.

### Chlorophyll a fluorescence

**Figure 5:**
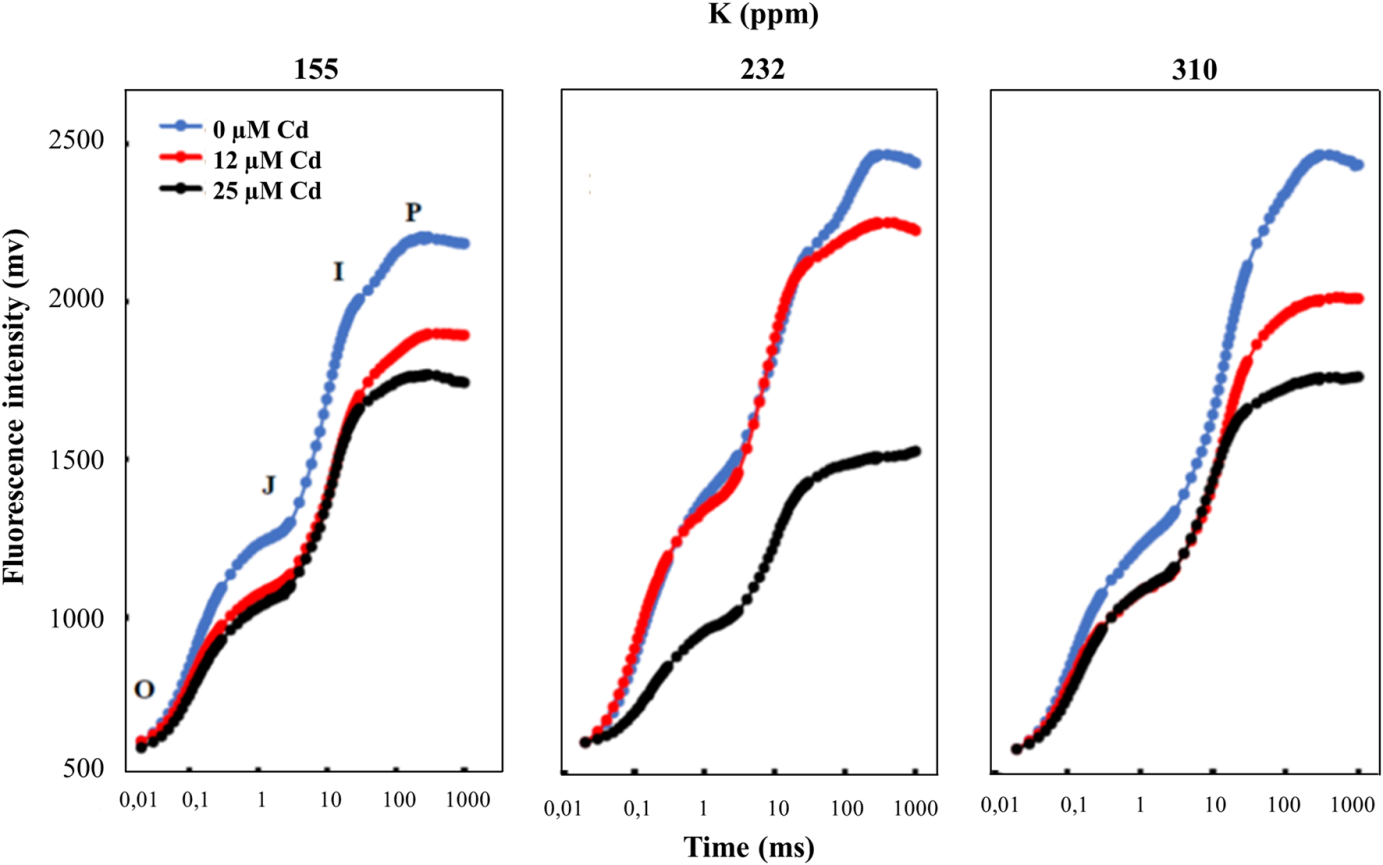
Effects of potassium concentration (155, 232, and 310 ppm) and three cadmium concentrations (0, 12 and 25 µM CdCl_2_) on Chlorophyll a fluorescence transient curve of tomato plants. Each value represents the mean ± SD of six independent repetitions, dissimilar letters indicate significant differences at *p* < 0.05 according to Student–Newman–Keuls test.

The plant grown in culture medium without Cd showed a typical ChlF OJIP transient (Fig. 5). The first O–J phase represents the reduction of the acceptor side of PS II^34^ and J–I phase represents the reduction/oxidation of the plastoquinone (PQ)^37–39^ and the last phase I–P represents the re-reduction of plastocyanin (PC)^+^ and P700^+^ in PS I^39, 40^. However, the fluorescence yield in plants grown in culture medium deficient in K seems to be affected. The concentration of 25 µM Cd showed visual changes and negative effects on the OJIP phases and more particularly the IP phase in addition to a decrease in the yield of chlorophyll fluorescence. Indeed, the Cd decreased the amplitude IP in comparison to treatment without Cd. Also, we noticed that the presence of 310 ppm of potassium in the culture medium of plants improved the yield of the ChlF in the presence of 25 µM Cd.

### Chlorophyll a fluorescence parameters

**Figure 6:**
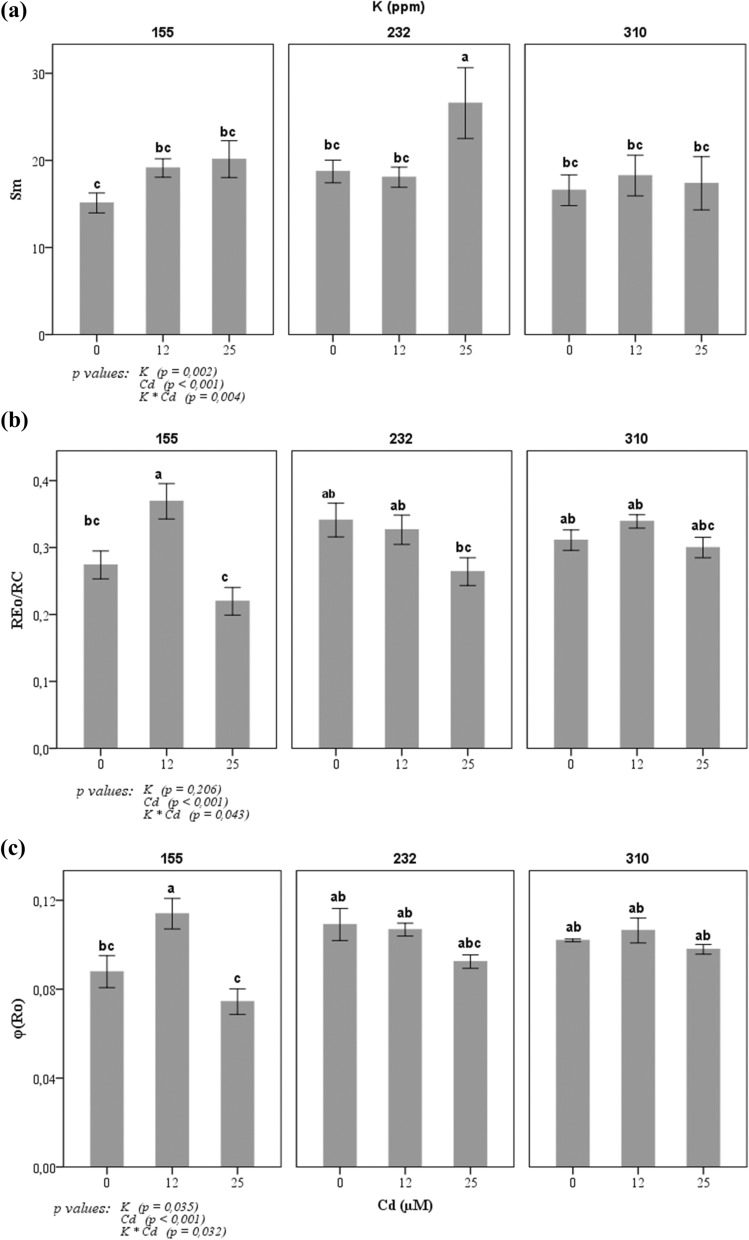
Effects of potassium concentration (155, 232, and 310 ppm) and three cadmium concentrations (0, 12 and 25 µM CdCl_2_) on Chlorophyll a fluorescence parameters of tomato plants. (**a**) Sm is a function of the number of electrons transported by PS II in the time range from 0 to tF_M_, the time to reach the maximum fluorescence intensity. (**b**) φ_Ro_ is the efficiency with which an electron can move from the reduced intersystem electron acceptors to the PSI end electron acceptors. (**c**) RE_o_/RC expressed the electron transport from Q_A_^−^ to the PSI electron acceptors. Each value represents the mean ± SD of six independent repetitions, dissimilar letters indicate significant differences at *p* < 0.05 according to Student–Newman–Keuls test.

The normalized area (Sm) is a measure of the energy needed to close all reaction centers^34^. In this work, the variation of this parameter indicates that the tomato plants respond differently to different concentrations of K and Cd (Fig. 6). In the culture medium with 155 or 232 ppm K in the presence of Cd, Sm increased significantly in the presence of 25 µM Cd compared to the treatment with 0 ppm Cd. Also, in the culture medium with 310 ppm K, we do not observe a variation of this parameter in plants. The same effect of all treatments was observed on the electron transport from Q_A_^−^ to the PSI electron acceptors RE/RC, φ_Ro_ (RE/ABS). These parameters varied in Cd-stressed plant grown in 155 and 232 ppm K. However, at 310 ppm K, the effect on the electron transport from Q_A_^−^ to the PSI electron acceptors is improved in presence of Cd.

## Discussion

In this work, the effect of Cd and K interaction on the physiology of tomato plants grown in a hydroponic medium has been investigated. It was demonstrated that the low concentration of K causes a decrease in the studied physiological parameters (Figs. 1, 2, 3, 4, 5, 6). This confirms the role of K in the plant growth and maintenance of cellular functionalities. The effect of low K appeared clearly in the decrease of chlorophyll content index (CCI), and roots and shoots dry weight compared to the other concentrations of K. Although plant responses to K deficiencies are well documented at the physiological level^41^ (and references therein). It is known that K plays a primordial role of chlorophyll in cellular physiology and mainly in the assurance of photosynthetic activity in plants^25, 30^. Tanaka and Tsuji^42^ reported that K^+^ prevents decomposition of formed chlorophyll and then plays a significant role in the formation of photosynthetic pigment.

The effect of Cd on cellular functioning is well established and it has been shown that Cd stress induces an imbalance in cell redox homeostasis leading to oxidative damage in plants^24, 43–46^. However, the interaction of Cd with the other elements remains an area of investigation. Here, the reduction of growth traits in presence of Cd (CCI, dry weights, root diameters and root surface area) may be due to the results of alteration of photosynthesis activity, and imbalance in nutrients uptake (Data not shown). Here, the figures presented in this work clearly showed that K mitigates the adverse effect of Cd. Indeed, we have demonstrated that the effect of Cd was mitigated by the high concentration of K (310 ppm) at physiological level. This has been observed by the improvement in the synthesis of chlorophyll (CCI), enhancing the dry weight of the roots and shoot and reduced Cd translocation. Shamsi et al.^47^ reported also that K supplementation alleviated the reduction of growth, photosynthesis and nutrients uptake in Cd-treated soybean. The ameliorating effect of the interaction between high K concentration and Cd is observed on two physiological parameters of the root morphology (root diameter and root surface area). Potassium supplementation confers plant exposed to Cd a positive response. This investigation suggests that K can efficiently reduce Cd-toxicity and improve health of plant by enhancing photosynthesis activity and the biosynthesis of photosynthetic pigments. According to previous studies, K plays significant regulatory roles in stomatal regulation, and energy transfer. These roles help plants to maintain their ion homeostasis, membrane integrity and antioxidant complex system activity. Furthermore, in our study, K plays a significant role in maintaining chlorophyll fluorescence in plants treated with Cd.

In addition to the positive effect of K at a high concentration and Cd on CCI and the dry weight of plants, we also observed that this interaction showed an improvement in the measured photosynthetic activity estimated here by chlorophyll fluorescence yield. We suppose that the Cd effect took place mainly at the level of the PSI, however, K seems to improve the electron transport around the PSI indicated by the increase in the amplitude of the I–P phase. The Cd translocation factor shown in Fig. 4 showed that K at a concentration of 310 ppm significantly reduced Cd translocation from root to shoot in tomato grown in hydroponic conditions. According to previous studies, cadmium influence several components of photosystem II. The main target on PSII is observed in an increase of inactive RCs. This reduction in active RCs leads induced also an increase of numbers of RC turnovers in electron transfer chain, thus, transforming the excitation energy into heat energy and can be an indication of photoinhibition^48, 49^. These finding is in accordance with our results on change of Sm fluorescence parameter that indicates the number of electrons transported by PSII in the time range from 0 to tFM. Indeed, supply of K can maintain the part of active RCs and then ensure a full reduction of the plastoquinone pool (see Fig. 6). The change of Chl a fluorescence transients shape of tomato leaves by Cd treatment suggested that Cd significantly influenced the electron transfer chain. Additionally, the method of chlorophyll fluorescence and OJIP-test may be used as a tool to understand the primary mode of action of heavy metals on the photosynthetic apparatus of plants. Previous research has suggested that Cd could exert multiple effects on both donor and acceptor sides of the PSII^50–52^. Cd could exchange with Ca^2+^ in oxygen-evolving complex on the donor side and decrease the rate of electron transfer from Q_A_ to Q_B_ due to interaction with non-heme Fe and conformational modification of Q_B_ pocket^53^. Previous studies reported also that Cd induced overproduction of reactive oxygen species (ROS) such as H_2_O_2_, O_2_^−^ and OH^−^ in cells^46, 54–57^. This overproduction of ROS altered photosynthesis activity which affects chlorophyll a fluorescence yield and other physiological and biochemical process^58–61^. In our study K supplementation may encounter the negative effect of ROS by enhancing antioxidant enzymes activities and then improved the tolerance response of tomato plant to Cd. Indeed, in another abiotic stress, K supplementation improved photosynthetic activity and transport of photosynthates and inhibition of ROS formation^62, 63^.

## Conclusion

We have shown that K plays an important role in the mitigation of Cd negative effects on physiological parameters of tomato plants grown in hydroponic conditions. Therefore, and in this context, optimization of K nutrition has shown promising results. The amelioration of the Cd effect by the addition of K may be associated with reduced Cd absorption. However, further study on Cd and essential nutrients interactions at the membrane level is necessary to understand the behavior of Cd uptake by plants. Unravel these interactions will allow us to significantly decrease bioaccumulation in different parts of the plant.
